# Supplementary figures and images for: Distinct trafficking routes of polarized and non-polarized membrane cargoes in Aspergillus nidulans
Source: eLife. 2024 Oct 21;13:e103355. doi: 10.7554/eLife.103355 (PMC11578586; doi:10.7554/eLife.103355)

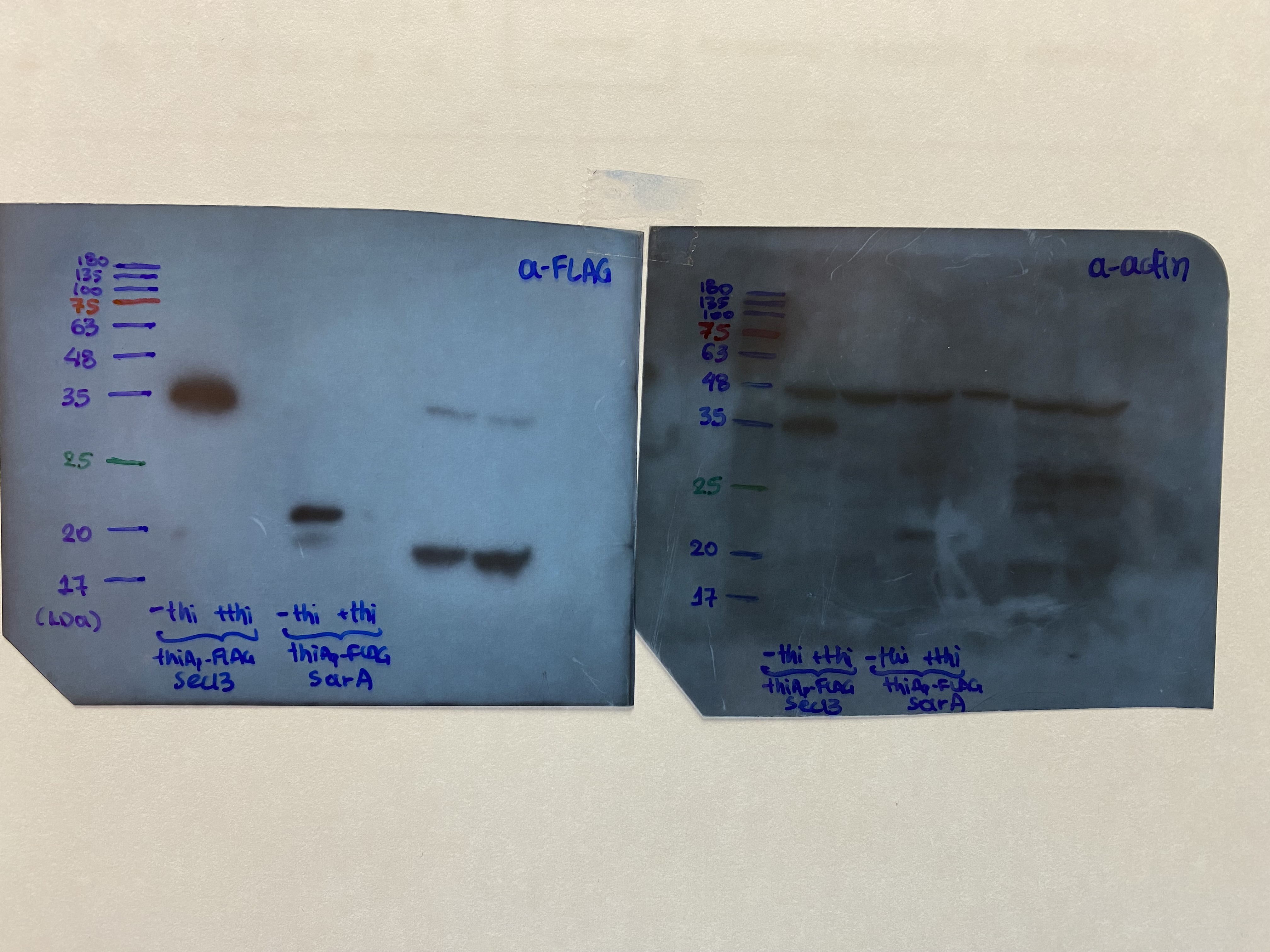

Supplement: Figure 4—source data 1. [file elife-103355-fig4-data1.zip › sec13 sarA.jpg]

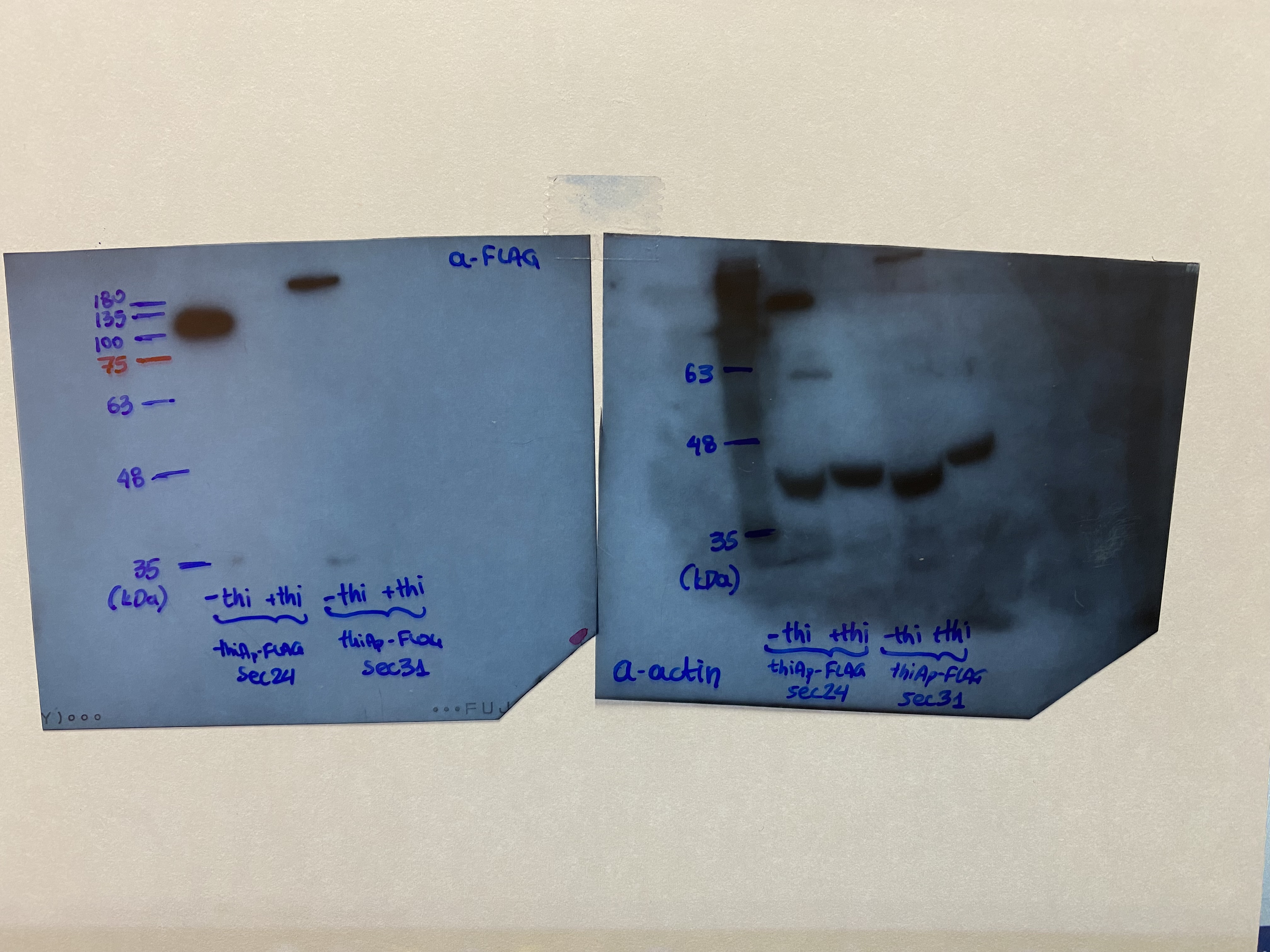

Supplement: Figure 4—source data 1. [file elife-103355-fig4-data1.zip › sec24 sec31.jpg]

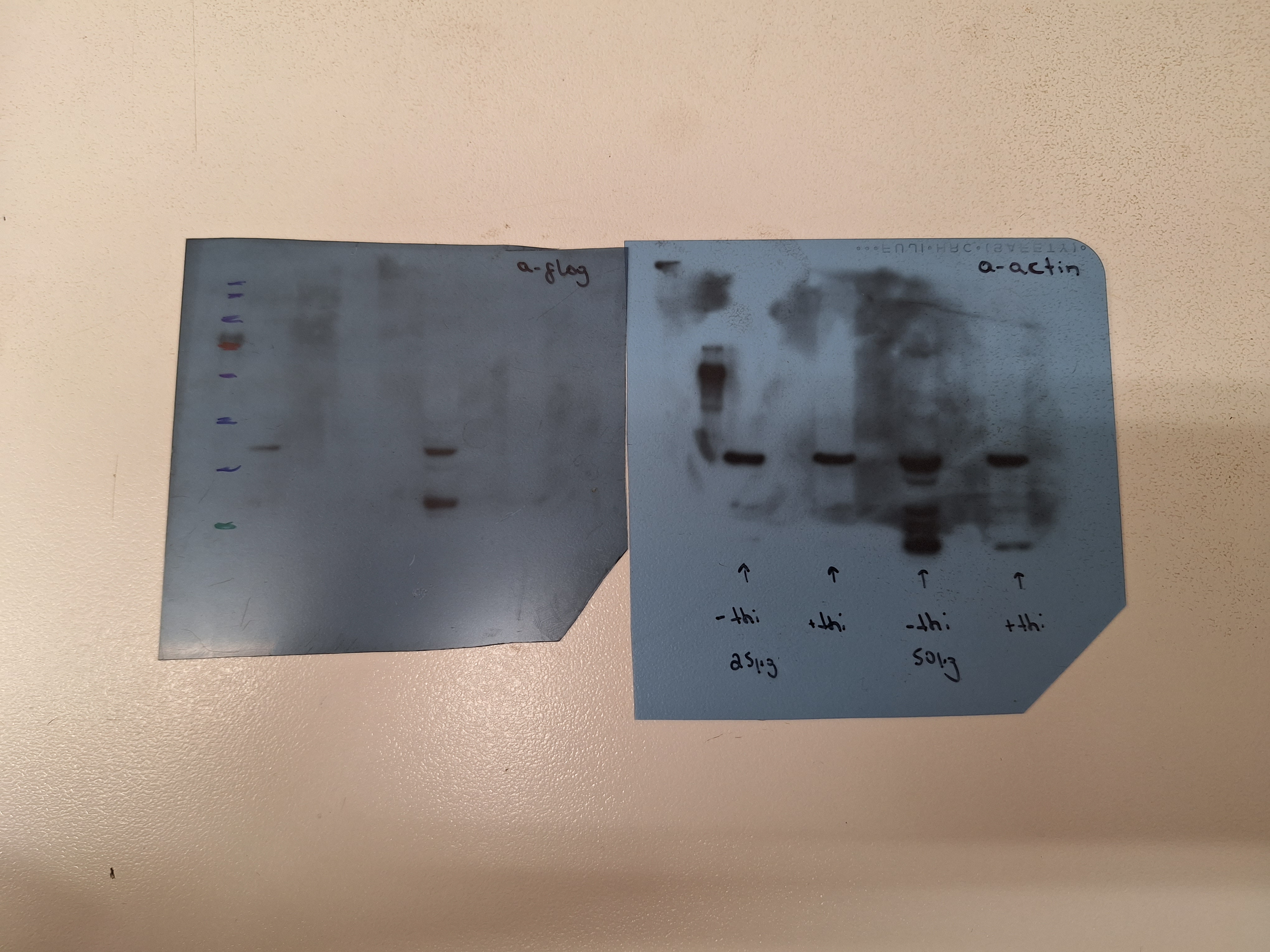

Supplement: Figure 8—source data 1. [file elife-103355-fig8-data1.jpg]
