# Supplementary material for: Distinct trafficking routes of polarized and non-polarized membrane cargoes in Aspergillus nidulans
Source: eLife. 2024 Oct 21;13:e103355. doi: 10.7554/eLife.103355 (PMC11578586; doi:10.7554/eLife.103355)
Supplement: Figure 4—source data 2. [file elife-103355-fig4-data2.pdf]

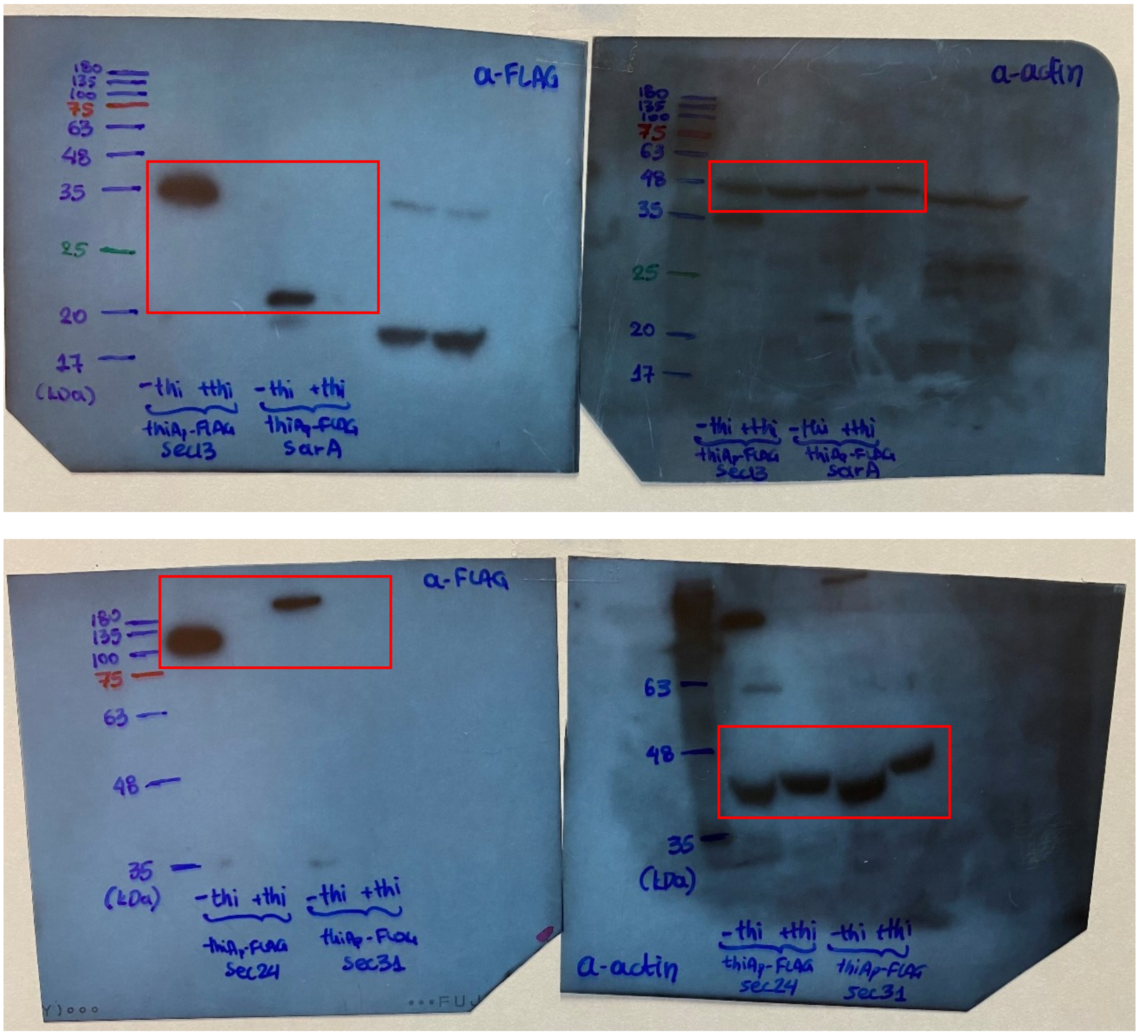

Figure 4 – Source data 1. Original X-Ray films corresponding to Figure 4, panel A. Molecular weight markers are shown in the left part of each film. The strains and the conditions used for growth are also shown (lower part of each film). The unspecific bands in the right part of the upper films correspond to the *thiA$_p$-FLAG-sec12* strain (not shown in Figure 4 due to inability for detection in western blots).
