# Supplementary material for: Distinct trafficking routes of polarized and non-polarized membrane cargoes in Aspergillus nidulans
Source: eLife. 2024 Oct 21;13:e103355. doi: 10.7554/eLife.103355 (PMC11578586; doi:10.7554/eLife.103355)
Supplement: Figure 8—source data 2. [file elife-103355-fig8-data2.pdf]

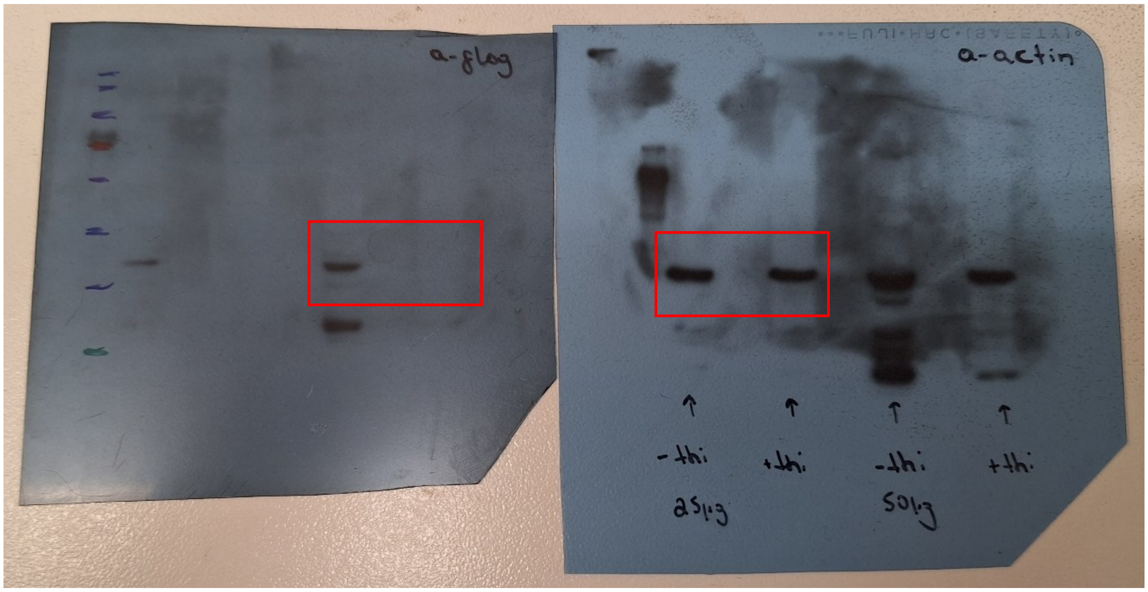

Figure 8 – Source data 1. Original X-Ray films corresponding to Figure 8, panel B. Molecular weight marker is shown in the left part. The conditions and the protein concentrations tested for the *thiA*$_p$*-FLAG-sec9* strain are shown in the lower part of the right film.
