# Supplementary material for: Distinct trafficking routes of polarized and non-polarized membrane cargoes in Aspergillus nidulans
Source: eLife. 2024 Oct 21;13:e103355. doi: 10.7554/eLife.103355 (PMC11578586; doi:10.7554/eLife.103355)
Supplement: Supplementary file 1. [file elife-103355-supp1.docx]

**Supplementary file 1. Strains used in this study**

All strains carry the *veA1* mutation affecting sporulation. *pabaA1, pyroA4, riboB2, argB2, pyrG89, and inoB2* are auxotrophic mutations for p-aminobenzoic acid, pyridoxine, riboflavin, arginine, uracil/uridine and inositol respectively. *wA4* is a mutation resulting in white conidiospore color.

| **Name** | **Genotype** | **Reference** |
| --- | --- | --- |
| TNO2A7 | *ΔnkuA::argB pyrG89 pyroA4 riboB2* | Nayak et al., 2006 |
| wt | *pabaA1* | wild-type reference strain |
| *alcA_p_-uapA-gfp alcA_p_-mCherry-synA* | *ΔuapA::alcA_p_-uapA-GFP::AFriboB alcA_p_-mCherry-synA::AFpyroA ΔnkuA::argB riboB2 pyroA4 pabaA1* | This study |
| Δ*uapA* | *ΔuapA riboB2* | Pantazopoulou et al., 2009 |
| Δ*synA* | *ΔsynAΔ::AFriboB ΔnkuA::argB pyrG89 riboB2 pyroA4* | This study |
| *alcA_p_-uapA-gfp mCherry-sedV* | *pyroA::gpdA^m^_p_::mCherry-sedV ΔuapA::alcA_p_-uapA-gfp::AFriboB pabaA1 inoB2* | Dimou et al., 2020 |
| *alcA_p_-uapA-gfp mRFP-PH^OSBP^* | *pyroA::gpdA^m^_p_::mRFP-PH^OSBP^ ΔuapA::alcA_p_-uapA-gfp::AFriboB pabaA1 inoB2* | Dimou et al., 2020 |
| *alcA_p_-gfp-synA mCherry-sedV* | *pyroA::gpdA^m^_p_::mCherry-sedV alcA_p_-gfp-synA::AFpyrG wA4 inoB2* | Dimou et al., 2020 |
| *alcA_p_-gfp-synA mrfp-PH^OSBP^* | *pyroA::gpdA^m^_p_::mrfp-PH^OSBP^ alcA_p_-gfp-synA::AFpyrG wA4 inoB2* | Dimou et al., 2020 |
| *thiA_p_-sarA alcA_p_-uapA-gfp alcA_p_-mCherry-synA* | *ΔuapA::alcA_p_-uapA-gfp::AFriboB alcA_p_-mCherry-synA::AFpyroA thiA_p_-sarA::AFpyrG ΔnkuA::argB riboB2 pyroA4* | This study |
| *thiA_p_-sec12 alcA_p_-uapA-gfp alcA_p_-mCherry-synA* | *ΔuapA::alcA_p_-uapA-gfp::AFriboB alcA_p_-mCherry-synA::AFpyroA thiA_p_-sec12::AFriboB ΔnkuA::argB riboB2 pyroA4 pabaA1* | This study |
| *thiA_p_-sec24 alcA_p_-uapA-gfp alcA_p_-mCherry-synA* | *ΔuapA::alcA_p_-uapA-GFP::AFriboB alcA_p_-mCherry-synA::AFpyroA thiA_p_-sec24::AFpyrG ΔnkuA::argB riboB2 pyroA4* | This study |
| *thiA_p_-sec13 alcA_p_-uapA-gfp alcA_p_-mCherry-synA* | *ΔuapA::alcA_p_-uapA-GFP::AFriboB alcA_p_-mCherry-synA::AFpyroA thiA_p_-sec13::AFpyrG ΔnkuA::argB riboB2 pyroA4* | This study |
| *thiA_p_-sec31 alcA_p_-uapA-gfp alcA_p_-mCherry-synA* | *ΔuapA::alcA_p_-uapA-GFP::AFriboB alcA_p_-mCherry-synA::AFpyroA thiA_p_-sec31::pabaA ΔnkuA::argB riboB2 pyroA4* | This study |
| *thiA_p_^FLAG^-sarA* | *thiA_p_^FLAG^-sarA::AFpyrG ΔnkuA::argB pyrG89 pyroA4 riboB2* | This study |
| *thiA_p_^FLAG^-sec12* | *thiA_p_^FLAG^-sec12::AFpyrG ΔnkuA::argB pyrG89 pyroA4 riboB2* | This study |
| *thiA_p_^FLAG^-sec24* | *thiA_p_^FLAG^-sec24::AFpyrG ΔnkuA::argB pyrG89 pyroA4 riboB2* | This study |
| *thiA_p_^FLAG^-sec13* | *thiA_p_^FLAG^-sec13::AFpyrG ΔnkuA::argB pyrG89 pyroA4 riboB2* | This study |
| *thiA_p_^FLAG^-sec31* | *thiA_p_^FLAG^-sec31::AFpyrG ΔnkuA::argB pyrG89 pyroA4 riboB2* | This study |
| *sec31^ts^-AV* | *sec31A1249V::AFpyrG ΔnkuA::argB pyrG89 riboB2 pyroA4* | This study |
| *sec31^ts^-AS* | *sec31A1249S::AFpyrG ΔnkuA::argB pyrG89 riboB2 pyroA4* | This study |
| *sec31^ts^-AG* | *sec31A1249G::AFpyrG ΔnkuA::argB pyrG89 riboB2 pyroA4* | This study |
| *sec31^ts^-AP* | *sec31A1249P::AFpyrG ΔnkuA::argB pyrG89 riboB2 pyroA4* | This study |
| *sec31^ts^-AP alcA_p_-uapA-gfp alcA_p_-mCherry-synA* | *sec31A1249P::AFpyrG ΔuapA::alcA_p_-uapA-gfp::AFriboB alcA_p_-mCherry-synA::AFpyroA ΔnkuA::argB riboB2 pyroA4* | This study |
| *sec31^ts^-AP alcA_p_-uapA-gfp sec16-mCherry* | *sec31A1249P::AFpyrG ΔuapA::alcA_p_-uapA-gfp::AFriboB sec16-mCheery::AFpyroA ΔnkuA::argB riboB2 pyroA4* | This study |
| *sec31^ts^-AP alcA_p_-gfp-synA sec16-mCherry* | *sec31A1249P::AFpyrG ΔuapA::alcA_p_-gfp-synA::AFriboB sec16-mCheery::AFpyroA ΔnkuA::argB riboB2 pyroA4* | This study |
| *thiA_p_-copA alcA_p_-uapA-gfp alcA_p_-mCherry-synA* | *ΔuapA::alcA_p_-uapA-gfp::AFriboB alcA_p_-mCherry-synA::AFpyroA thiA_p_-copA::AFpyrG ΔnkuA::argB riboB2 pyroA4* | This study |
| *thiA_p_-arfA alcA_p_-uapA-gfp alcA_p_-mCherry-synA* | *ΔuapA::alcA_p_-uapA-gfp::AFriboB alcA_p_-mCherry-synA::AFpyroA thiA_p_-arfA::AFpyrG ΔnkuA::argB riboB2 pyroA4* | This study |
| *thiA_p_-sedV alcA_p_-uapA-gfp alcA_p_-mCherry-synA* | *ΔuapA::alcA_p_-uapA-gfp::AFriboB alcA_p_-mCherry-synA::AFpyroA thiA_p_-sedV::AFpyrG ΔnkuA::argB riboB2 pyroA4 pabaA1* | This study |
| *thiA_p_-geaA alcA_p_-uapA-gfp alcA_p_-mCherry-synA* | *ΔuapA::alcA_p_-uapA-gfp::AFriboB alcA_p_-mCherry-synA::AFpyroA thiA_p_-geaA::AFpyrG ΔnkuA::argB riboB2 pyroA4 pabaA1* | This study |
| *thiA_p_-rabO alcA_p_-uapA-gfp alcA_p_-mCherry-synA* | *ΔuapA::alcA_p_-uapA-gfp::AFriboB alcA_p_-mCherry-synA::AFpyroA thiA_p_-rabO::AFpyrG ΔnkuA::argB riboB2 pyroA4 pabaA1* | This study |
| *thiA_p_-hypB alcA_p_-uapA-gfp alcA_p_-mCherry-synA* | *ΔuapA::alcA_p_-uapA-gfp::AFriboB alcA_p_-mCherry-synA::AFpyroA thiA_p_-hypB::AFpyrG ΔnkuA::argB riboB2 pyroA4* | This study |
| *thiA_p_-rabE alcA_p_-uapA-gfp alcA_p_-mCherry-synA* | *ΔuapA::alcA_p_-uapA-gfp::AFriboB alcA_p_-mCherry-synA::AFpyroA thiA_p_-rabE::AFpyrG ΔnkuA::argB riboB2 pyroA4* | This study |
| *thiA_p_-ap1^σ^ alcA_p_-uapA-gfp alcA_p_-mCherry-synA* | *ΔuapA::alcA_p_-uapA-gfp::AFriboB alcA_p_-mCherry-synA::AFpyroA thiA_p_-ap1^σ^::AFriboB ΔnkuA::argB riboB2 pyroA4 pabaA1* | This study |
| *alcA_p_-uapA-gfp mCherry-sedV thiA_p_-rabE* | *ΔuapA::alcA_p_-uapA-gfp::AFriboB pyroA::gpdA^m^_p_::mCherry-sedV thiA_p_-rabE::AFpyrG* | This study |
| *thiA_p_-rabE alcA_p_-uapA-gfp mRFP-PH^OSBP^* | *pyroA::gpdA^m^_p_::mRFP-PH^OSBP^ ΔuapA::alcA_p_-uapA-gfp::AFriboB thiA_p_-rabE::AFpyrG pabaA1* | This study |
| *thiA_p_-ykt6 alcA_p_-uapA-gfp alcA_p_-mCherry-synA* | *ΔuapA::alcA_p_-uapA-gfp::AFriboB alcA_p_-mCherry-synA::AFpyroA thiA_p_-ykt6::AFpyrG ΔnkuA::argB riboB2 pyroA4 pabaA1* | This study |
| *Δsec22 alcA_p_-uapA-gfp alcA_p_-mCherry-synA* | *ΔuapA::alcA_p_-uapA-gfp::AFriboB alcA_p_-mCherry-synA::AFpyroA Δsec22::AFpyrG ΔnkuA::argB riboB2 pyroA4 pabaA1* | This study |
| *thiA_p_-ykt6 Δsec22 alcA_p_-uapA-gfp alcA_p_-mCherry-synA* | *ΔuapA::alcA_p_-uapA-gfp::AFriboB alcA_p_-mCherry-synA::AFpyroA thiA_p_-ykt6::AFriboB Δsec22::AFpyrG ΔnkuA::argB riboB2 pyroA4* | This study |
| *thiA_p_-sft1 alcA_p_-uapA-gfp alcA_p_-mCherry-synA* | *ΔuapA::alcA_p_-uapA-gfp::AFriboB alcA_p_-mCherry-synA::AFpyroA thiA_p_-sft1::pabaA ΔnkuA::argB riboB2 pyroA* | This study |
| *thiA_p_-bos1 alcA_p_-uapA-gfp alcA_p_-mCherry-synA* | *ΔuapA::alcA_p_-uapA-gfp::AFriboB alcA_p_-mCherry-synA::AFpyroA thiA_p_-bos1::pabaA ΔnkuA::argB riboB2 pyroA4* | This study |
| *thiA_p_^FLAG^-ykt6* | *thiA_p_^FLAG^-ykt6::pyrG89 ΔnkuA::argB pyrG89 pyroA4 riboB2* | This study |
| *thiA_p_^FLAG^-sft1* | *thiA_p_^FLAG^-sft1::AFpyrG ΔnkuA::argB pyrG89 pyroA4 riboB2* | This study |
| *thiA_p_^FLAG^-bos1* | *thiA_p_^FLAG^-bos1::AFpyrG ΔnkuA::argB pyrG89 pyroA4 riboB2* | This study |
| *ΔrabD* | *ΔrabD::AFpyrG ΔnkuA::argB pyrG89 riboB2 pyroA4* | This study |
| *thiA_p_-ssoA* | *thiA_p_-ssoA::AFpyrG ΔnkuA::argB pyrG89 riboB2 pyroA4* | Dimou et al., 2020 |
| *thiA_p_-sec9* | *thiA_p_-sec9::AFpyrG ΔnkuA::argB pyrG89 riboB2 pyroA5* | This study |
| *thiA_p_^FLAG^-ssoA* | *thiA_p_^FLAG^-ssoA::AFpyrG ΔnkuA::argB pyrG89 pyroA4 riboB2* | This study |
| *thiA_p_^FLAG^-sec9* | *thiA_p_^FLAG^-sec9::AFpyrG ΔnkuA::argB pyrG89 pyroA4 riboB2* | This study |
| *uapA-gfp* | *ΔuapA::uapA-gfp::AFriboB ΔuapC::AfpyrG ΔnkuA::argB pabaA1 pyroA4 riboB2* | Evangelinos et al., 2016 |
| *thiAp-ssoA uapA-gfp* | *ΔuapA::uapA-gfp thiA_p_-ssoA::AFpyrG ΔnkuA::argB pabaA1* | Dimou et al., 2020 |
| *thiAp-sec9 uapA-gfp* | *ΔuapA::uapA-gfp thiA_p_-sec9::AFpyrG ΔnkuA::argB pabaA1* | This study |
| *ΔsynA uapA-gfp* | *ΔuapA::uapA-gfp ΔsynA:AFriboB ΔnkuA::argB pabaA1* | This study |
| *gfp-chsB* | *gfp-chsB::AFpyrG ΔnkuA::argB pyrG89 pyroA4 riboB2* | Dimou et al., 2020 |
| *thiAp-ssoA gfp-chsB* | *gfp-chsB::AFpyrG thiA_p_-ssoA::AFriboB ΔnkuA::argB pyrG89 pyroA4 riboB2* | This study |
| *thiAp-sec9 gfp-chsB* | *gfp-chsB::AFpyrG thiA_p_-sec9::AFpyrG pyrG89 pyroA4* | This study |
| *ΔsynA gfp-chsB* | *gfp-chsB::AFpyrG ΔsynA::AFriboB ΔnkuA::argB pyrG89 pyroA4 riboB2* | This study |
| *ΔrabD alcA_p_-uapA-gfp alcA_p_-mCherry-synA* | *ΔuapA::alcA_p_-uapA-gfp::AFriboB alcA_p_-mCherry-synA::AFpyroA ΔrabD::AFpyrG ΔnkuA::argB riboB2 pyroA4 pabaA1* | This study |
| *ΔnyvA* | *ΔnyvA::AFpyrG ΔnkuA::argB pyrG89 riboB2 pyroA4* | This study |
| *Δsec22 ΔnyvA ΔsynA* | *Δsec22::AFpyrG ΔnyvA::AFpyroA ΔsynA::AFriboB ΔnkuA::argB pyrG89 riboB2 pyroA4* | This study |
| *ΔnyvA uapA-gfp* | *ΔuapA::uapA-gfp ΔnyvA:AFpyrG ΔnkuA::argB pabaA1* | This study |
| *Δsec22 ΔnyvA ΔsynA uapA-gfp* | *ΔuapA::uapA-gfp Δsec22::AFpyrG ΔnyvA::AFpyrG ΔsynA::AFriboB ΔnkuA::argB pyroA4 pabaA1* | This study |
| *ΔnyvA gfp-chsB* | *gfp-chsB::AFpyrG ΔnyvA:AFpyrG pyrG89 pyroA4* | This study |
| *Δsec22 ΔnyvA ΔsynA gfp-chsB* | *gfp-chsB::AFpyrG Δsec22::AFpyrG ΔnyvA::AFpyrG ΔsynA::AFriboB ΔnkuA::argB pyroA4 pabaA1* | This study |
