# Supplementary material for: Distinct trafficking routes of polarized and non-polarized membrane cargoes in Aspergillus nidulans
Source: eLife. 2024 Oct 21;13:e103355. doi: 10.7554/eLife.103355 (PMC11578586; doi:10.7554/eLife.103355)
Supplement: Supplementary file 2. [file elife-103355-supp2.docx]

**Supplementary file 2. Annotation of proteins used in this study**

| **FungiDB ID** | **Systematic/Protein name** | **Trafficking process** |
| --- | --- | --- |
| AN0411 | SarA^Sar1^ | ER exit sites (ERes) |
| AN3720 | Sec24 | ER exit sites (ERes) |
| AN4317 | Sec13 | ER exit sites (ERes) |
| AN6257 | Sec31 | ER exit sites (ERes) |
| AN11127 | Sec12 | ER exit sites (ERes) |
| AN3026 | CopA^CopI^ | COPI coat |
| AN1126 | ArfA^Arf1/2^ | Early/late Golgi sorting |
| AN0112 | GeaA^Gea1/2^ | Early Golgi sorting |
| AN6709 | HypB^Sec7^ | Late Golgi sorting |
| AN4281 | RabO^Ypt1/Rab1^ | Early Golgi sorting |
| AN0347 | RabE^Ypt31/32/Rab11^ | Post-Golgi |
| AN7682 | Ap1_σ_^Aps1^ | Late Golgi sorting |
| AN6974 | RabD^Sec4^ | exocyst |
| AN8488 | Ykt6 | R-SNARE |
| AN8769 | SynA^Snc1/2^ | R-SNARE |
| AN0571 | NyvA^Nyv1^ | R-SNARE |
| ChrI_A_nidulans_FGSC_A4 2,596,682-2,597,694 | Sec22 | R-SNARE |
| AN3416 | SsoA^Sso1/2^ | Qa-SNARE |
| AN9526 | SedV^Sed5^ | Qa-SNARE |
| AN2419 | Sec9 | Qb-SNARE |
| AN11900 | Bos1 | Qb-SNARE |
| AN10508 | Sft1 | Qc-SNARE |
| AN2523 | ChsB^Chs3^ | apical cargo |
| AN6932 | UapA | non-polar cargo |
