# Supplementary material for: Distinct trafficking routes of polarized and non-polarized membrane cargoes in Aspergillus nidulans
Source: eLife. 2024 Oct 21;13:e103355. doi: 10.7554/eLife.103355 (PMC11578586; doi:10.7554/eLife.103355)
Supplement: Supplementary file 3. [file elife-103355-supp3.docx]

**Supplementary file 3. Primers used for cloning and gene construction**

| **Plasmids** | **Oligonucleotides** | **5’3’ Sequence** |
| --- | --- | --- |
| pGEM *sec31*A1249V::*AFpyrG* / pGEM *sec31*A1249P::*AFpyrG* / pGEM *sec31A1249S::AFpyrG* / pGEM *sec31A1249G::AFpyrG* | sec31 ORF SphI F | GCGGCATGCCGTCCAGTGCACCCATTTCCTC |
|  | sec31 3’ UTR XbaI R | CGCGTCTAGACGTAATTCCTCGGGAGCGCG |
|  | sec31 3’ XbaI F | CGCGTCTAGACGGATGAAACGACACTGTCGACG |
|  | sec31 3’ NotI R | CGCGCGGCCGCCGGCGATCGTTCAACACTGCTAG |
|  | AFpyrG XbaI F | CGCGTCTAGAGCCTCAAACAATGCTCTTCACCC |
|  | AFpyrG XbaI R | CGGTCTAGACTGTCTGAGAGGAGGCACTGATGCG |
|  | sec31 A1249V F | GCACGTGACTACGAGACGGTTCGTACAATCCACATTGATATCATGAC |
|  | sec31 A1249V R | GTCATGATATCAATGTGGATTGTACGAACCGTCTCGTAGTCACGTGC |
|  | sec31 A1249P F | GCACGTGACTACGAGACGCCTCGTACAATCCACATTGATATCATGAC |
|  | sec31 A1249P R | GTCATGATATCAATGTGGATTGTACGAGGCGTCTCGTAGTCACGTGC |
|  | sec31 A1249S F | GCACGTGACTACGAGACGAGTCGTACAATCCACATTGATATCATGAC |
|  | sec31 A1249S R | GTCATGATATCAATGTGGATTGTACGACTCGTCTCGTAGTCACGTGC |
|  | sec31 A1249G F | GCACGTGACTACGAGACGGGTCGTACAATCCACATTGATATCATGAC |
|  | sec31 A1249G R | GTCATGATATCAATGTGGATTGTACGACCCGTCTCGTAGTCACGTGC |
|  | sec31 3’ check R | CCGAACACAGAGAGCCGAGG |
|  | sec31 seq F | GGCCAAGCACAGCCTCTTCTG |
| pGEM *sec16-mCherry* | sec16-ORF-AatII-F | CGCGGACGTCGGTCGTGGATTTCGAAACCAAGCATG |
|  | sec16-ORFns-SpeI-R | CGCGACTAGTTTGGGCCATTACATCAACATACCGGC |
|  | sec16-3-SpeI-F | CGCGACTAGTGAAGCTCTTAACATCGCTTCCAACTC |
|  | sec16-3-NotI-R | CGCGGCGGCCGCGAGGTCCCGCTTCCTGCACTTCAC |
|  | mCherry-SpeI-F | GCGACTAGTGGAGCAGGTGCTGGTGCTG |
|  | mCherry-XbaI-R-3 | CGCGTCTAGACCTGTTATCCCTAGCGGATCTG |
| pGEM *thiA_p-_ykt6::AFpyrG* / pGEM *thiA_p-_ykt6::AFriboB* | ykt6 5’ ApaI F | CGCGGGGCCCCTGCAATTGCCTGCATCTGTGCTG |
|  | ykt6 5’ SpeI R | CGCGACTAGTGCTGGATGAGGCAGGGAGATAATAG |
|  | ykt6 ORF SpeI F | CGCGACTAGTATGAAGATCGTTTACATTGGTGTAAGCTGC |
|  | ykt6 ORF NotI R | CGCGGCGGCCGCCTACTACTATGGTTGGCGGGATACGG |
|  | AFpyrG SpeI F | CGCGACTAGTGCCTCAAACAATGCTCTTCACCCTC |
|  | AFriboB SpeI F | CGCGACTAGTCCCGGGCTGCAGGAATTCG |
|  | thiAp SpeI R | CGCGACTAGTGTTGACTCAGTTCAATGGTTCGAC |
| pGEM Δ*sec22*::*AFpyrG* | sec22 5’ SphI F | CGCGGCATGCGTGCTGTAGGCCCTTGAATGTCC |
|  | sec22 5’ SpeI R | CGCGACTAGTGTTATAGCTGGGTTCGTTAACTGGCGG |
|  | sec22 3’ SpeI F | CGCGACTAGT CCCAGCGATGCCACGTAACTACC |
|  | sec22 3’ NotI R | CGCGGCGGCCGCGGCTCCTTCCAAGTTGTCTCATCG |
|  | AFpyrG SpeI F | CGCGACTAGTGCCTCAAACAATGCTCTTCACCCTC |
|  | AFpyrG SpeI R | CGGACTAGTCTGTCTGACAGGAGGCACTGATGCG |
| pGEM *thiA_p-_sec9::AFpyrG* / pGEM *thiA_p-_FLAG-sec9::AFpyrG* | sec9 5’ SphI F | GCGGCATGCCGGAGTTGACTGAACAGTTGGTCAG |
|  | sec9 5’ SpeI R | CGACTAGTCGACTCCACTAAGCCGAAAGAAGC |
|  | sec9 ORF SpeI F | GCGACTAGTATGAAGCGATTCGGCCTCAAAAAGTC |
|  | sec9 ORF NotI R | CGCGCGGCCGCGCCAGGCAGTCGTCATAACAATCG |
|  | AFpyrG XbaI F | CGCGTCTAGAGCCTCAAACAATGCTCTTCACCC |
|  | AFpyrG SpeI R | CGGACTAGTCTGTCTGACAGGAGGCACTGATGCG |
|  | thiAp SpeI F | CGCGACTAGTCGACCTGGCACCTACAGAAGAATC |
|  | thiAp SpeI R | CGCGACTAGTGTTGACTCAGTTCAATGGTTCGAC |
|  | thiApFLAG XbaI R | CGCGTCTAGACTTGTCATCGTCGTCCTTGTAGTCC |
| pGEM Δ*synA*::*AFriboB* | synA 5’ ApaI F | CGCGGGGCCCGACTTGCCGTGATCATATATCTTGTCC |
|  | synA 5’ SpeI R | CGCGACTAGTCACGGGTTGTGGAAGAAGAGGAGG |
|  | synA 3’ SpeI F | CGCGACTAGTCTACGACACTTCAACGACGGTCATG |
|  | synA 3’ NotI R | CGCGGCGGCCGCGACTTGGTGGTGGAAGCTCTCCG |
|  | AFriboB SpeI F | CGCGACTAGTCCCGGGCTGCAGGAATTCG |
|  | AFriboB SpeI R | CGCGACTAGTCCCGGGCTGCAGGAATTCGA |
| pGEM *ΔnyvA::AFpyrG /* pGEM *ΔnyvA::AFpyroA* | nyvA 5’ ApaI F | CGCGGGGCCCGCTGGGTTCGTTGGATGATGAC |
|  | nyvA 5’ XbaI R | CGCGTCTAGAGCCAAAATACCAAGAGCCAACCG |
|  | nyvA 3’ XbaI F | CGCGTCTAGAGGCCATATATGTGCAATTGTGTCAC |
|  | nyvA 3’ NotI R | CGCGCGGCCGCGTCATGTGACGCACATTCCACTG |
|  | AFpyrG XbaI F | CGCGTCTAGAGCCTCAAACAATGCTCTTCACCC |
|  | AFpyrG XbaI R | CGGTCTAGACTGTCTGAGAGGAGGCACTGATGCG |
|  | AFpyroA SpeI F | CGCGACTAGTGGACATCAGATGCTGGATTAC |
|  | AFpyroA SpeI R | CGCGACTAGTGCGAGTGTCTACATAATGAAGG |
| pGEM *alcA_p-_mCherry-synA::AFpyroA* | AFpyroA XbaI F | CGCGTCTAGAGGACATCAGATGCTGGATTAC |
|  | AFpyroA SpeI R | CGCGACTAGTGCGAGTGTCTACATAATGAAGG |
|  | alcAp XbaI F | CGCTCTAGATAAGTCCCTTCGTATTTCTCCGC |
|  | alcAp XbaI R | CGCGTCTAGAATTTTGAGGCGAGGTGATAGG |
|  | mCherry ORF SpeI F | GCGCACTAGTATGGTGAGCAAGGGCGAGG |
|  | mCherry NS GA XbaI R | GCGCTCTAGATGCTCCCTTGTACAGCTCGTCCATGCC |
|  | 5 AN8769 ApaI F | GCGCGGGCCCCCTGCGAGATCCAATGACTC |
|  | 5 AN8769 SpeI R | GCGCACTAGTGATGCTCTGTGCGAAGAGCTTG |
|  | AN8769 5 check | CTGATGAATTCAACCGTAGTGTCCG |
|  | AN8769 ORF SpeI F | CGCGACTAGTATGTCTGAGCAACCGTACGATCC |
|  | AN8769 ORF3 NotI R | CGCGGCGGCCGCGTGACTCAGACCACCAAGATCACC |
| pGEM *thiA_p_-FLAG-sarA::AFpyrG* | AN0411 5 ApaI F | CGCGGGGCCCGAAGACTCGGAAGCAACTAGGTTTC |
|  | AN0411 5 SpeI R | CGCGACTAGTCGCCGTGTAAATGCAGACACAAAG |
|  | AN0411 ORF SpeI F | CGCGACTAGTATGTGGATTATCAACTGGTGTAAGTCTGC |
|  | AN0411 3 NotI R | CGCGCGGCCGCGGAGCTTAACCAGAATGCCAGTG |
|  | AFpyrG XbaI F | CGCGTCTAGAGCCTCAAACAATGCTCTTCACCCTC |
|  | AFpyrG SpeI R | CGGACTAGTCTGTCTGAGAGGAGGCACTGATGCG |
|  | thiAp XbaI F | CGCGTCTAGACGACCTGGCACCTACAGAAGAATC |
|  | thiA FLAG XbaI R | CGCGTCTAGACTTGTCATCGTCGTCCTTGTAGTCCATGTTGACTCAGTTCAATGGTTCGACTATAG |
| pGEM *thiA_p_-FLAG-sec12::AFpyrG* | AN11127 5 ApaI F | CGCGGGGCCCGCGTGTCCGAGAATTTCTGATGG |
|  | AN11127 5 XbaI R | CGCGTCTAGACCAGGGGTGTGACAACTAATTGC |
|  | AN11127 5 check | GCCAAATACGACATCAGAAACCC |
|  | AN11127 ORF XbaI F | CGCGTCTAGAATGGCGCCCAAAATACCGTCTGC |
|  | AN11127 ORF NotI R | CGCGGCGGCCGCCAAGATACGGAGGCATGACGCCTC |
|  | AFpyrG XbaI F | CGCGTCTAGAGCCTCAAACAATGCTCTTCACCCTC |
|  | AFpyrG SpeI R | CGGACTAGTCTGTCTGAGAGGAGGCACTGATGCG |
|  | thiAp XbaI F | CGCGTCTAGACGACCTGGCACCTACAGAAGAATC |
|  | thiA FLAG XbaI R | CGCGTCTAGACTTGTCATCGTCGTCCTTGTAGTCCATGTTGACTCAGTTCAATGGTTCGACTATAG |
| pGEM *thiA_p_-FLAG-sec24::AFpyrG* | AN3720 5 SphI F | CGCGGCATGCCGGCTCAAACAAGTCGAAGACCTTATC |
|  | AN3720 5 SpeI R | CGCGACTAGTCTAGGCATTTGCAGCTTATGAACGTTTC |
|  | AN3720 ORF SpeI F | CGCGACTAGTATGGCATCTCCACAAGGGGGCTAC |
|  | AN3720 ORF NotI R | CGCGGCGGCCGCCACAGACACTTGTGCCTTGGAGCAC |
|  | AFpyrG XbaI F | CGCGTCTAGAGCCTCAAACAATGCTCTTCACCCTC |
|  | AFpyrG SpeI R | CGGACTAGTCTGTCTGAGAGGAGGCACTGATGCG |
|  | thiAp XbaI F | CGCGTCTAGACGACCTGGCACCTACAGAAGAATC |
|  | thiA FLAG XbaI R | CGCGTCTAGACTTGTCATCGTCGTCCTTGTAGTCCATGTTGACTCAGTTCAATGGTTCGACTATAG |
| pGEM *thiA_p_-FLAG-sec13::AFpyrG* | AN4317 5 ApaI F | CGCGGGGCCCCCAGCTTGCGTACATCGAATCATG |
|  | AN4317 5 SpeI R2 | CGCGACTAGTGAGCTATTTGCAGCTCTAGGCCGATG |
|  | AN4317 ORF SpeI F | CGCGACTAGTATGGTACGTCTTCGCCGTCAATTC |
|  | AN4317 3 NotI R | CGCGCGGCCGCGGAAGACAGGATTTGCTGGCTTG |
|  | AFpyrG XbaI F | CGCGTCTAGAGCCTCAAACAATGCTCTTCACCCTC |
|  | AFpyrG SpeI R | CGGACTAGTCTGTCTGAGAGGAGGCACTGATGCG |
|  | thiAp XbaI F | CGCGTCTAGACGACCTGGCACCTACAGAAGAATC |
|  | thiA FLAG XbaI R | CGCGTCTAGACTTGTCATCGTCGTCCTTGTAGTCCATGTTGACTCAGTTCAATGGTTCGACTATAG |
| pGEM *thiA_p_-sec31::pabaA*/ pGEM *thiA_p_-FLAG-sec31::AFpyrG* | 5 sec31 ApaI F | GCGCGGGCCCGTACGTCCGTACAGCTCGCTC |
|  | 5 sec31 SpeI R | GCGCACTAGTGCTGAGAGTAGGGGTCTGC |
|  | ORF sec31 SpeI F | GCGCACTAGTATGGTGCGTCTGAGGGAGATTC |
|  | ORFsec31NotIR | GCGCGCGGCCGCCCTGGGCAAATAATCAACAGTTTC |
|  | AFpyrG XbaI F | CGCGTCTAGAGCCTCAAACAATGCTCTTCACCCTC |
|  | AFpyrG SpeI R | CGGACTAGTCTGTCTGAGAGGAGGCACTGATGCG |
|  | thiAp XbaI F | CGCGTCTAGACGACCTGGCACCTACAGAAGAATC |
|  | thiA FLAG XbaI R | CGCGTCTAGACTTGTCATCGTCGTCCTTGTAGTCCATGTTGACTCAGTTCAATGGTTCGACTATAG |
|  | thiA SpeI F | CGCGACTAGTCGACCTGGCACCTACAGAAGAATC |
|  | thiA XbaI R | CGCGTCTAGAGTTGACTCAGTTCAATGGTTCGAC |
| pGEM *thiA_p_-FLAG-ykt6::AFpyrG* | AN8488 5 ApaI F | CGCGGGGCCCCTGCAATTGCCTGCATCTGTGCTG |
|  | AN8488 5 SpeI R | CGCGACTAGTGCTGGATGAGGCAGGGAGATAATAG |
|  | AN8488 ORF SpeI F | CGCGACTAGTATGAAGATCGTTTACATTGGTGTAAGCTGC |
|  | AN8488 ORF NotI R | CGCGGCGGCCGCCTACTACTATGGTTGGCGGGATACGG |
|  | AFpyrG XbaI F | CGCGTCTAGAGCCTCAAACAATGCTCTTCACCCTC |
|  | AFpyrG SpeI R | CGGACTAGTCTGTCTGAGAGGAGGCACTGATGCG |
|  | thiAp XbaI F | CGCGTCTAGACGACCTGGCACCTACAGAAGAATC |
|  | thiA FLAG XbaI R | CGCGTCTAGACTTGTCATCGTCGTCCTTGTAGTCCATGTTGACTCAGTTCAATGGTTCGACTATAG |
| pGEM *thiA_p_-FLAG-ssoA::AFpyrG* | AN3416 5 ApaI F | CGCGGGGCCCGTGTGTTTTCCCAGGCTCTGGGC |
|  | AN3416 5 SpeI R | CGCGACTAGTCAGCTCCTGACGACAGCAGTGAC |
|  | AN3416 ORF SpeI F | CGCGACTAGTATGAGTGTATGTCCGCCGGCCTAATG |
|  | AN3416 3 NotI R | CGCGGCGGCCGCCGTCAAGCCGCCTCATAGATGCAAC |
|  | AFpyrG XbaI F | CGCGTCTAGAGCCTCAAACAATGCTCTTCACCCTC |
|  | AFpyrG SpeI R | CGGACTAGTCTGTCTGAGAGGAGGCACTGATGCG |
|  | thiAp XbaI F | CGCGTCTAGACGACCTGGCACCTACAGAAGAATC |
|  | thiA FLAG XbaI R | CGCGTCTAGACTTGTCATCGTCGTCCTTGTAGTCCATGTTGACTCAGTTCAATGGTTCGACTATAG |
| pGEM Δ*rabD*::*AFpyrG* | AN6974 5 ApaI F | CGCGGGGCCCGACCTACGAAGTCCTTTTATGGGC |
|  | AN6974 5 SpeI R | CTATAATCAGCCGACTAGTGGTTCG |
|  | AN6974 3 NotI R | CGCGGCGGCCGCCTCCGGCGTTTCTACCGATTG |
|  | AN6974 3 SpeI F | GGGACTAGTATGTGTATTCTCCTG |
|  | AN6974 5 check | GAAACTTGTCCGGACCATACGCC |
|  | AFpyrG SpeI F | CGCGACTAGTGCCTCAAACAATGCTCTTCACCCTC |
|  | AFpyrG SpeI R | CGGACTAGTCTGTCTGACAGGAGGCACTGATGCG |
| pGEM *thiA_p_-rabO::AFpyrG* | rabO 5 ApaI F | CGCGGGGCCCGAAGGGTTCAAGGATGAAACGAAC |
|  | rabO 5 XbaI R | CGCGTCTAGACTCAGTGCAGGAGAGCAAAGGAG |
|  | rabO ORF XbaI F | CGCGTCTAGAATGAACCCTGAGTGGTAAGTGCTTTG |
|  | rabO 3 NotI R | CGCGGCGGCCGCCAACCTCTAATTTACCACGGCAGC |
|  | AFpyrG SpeI F | CGCGACTAGTGCCTCAAACAATGCTCTTCACCCTC |
|  | AFpyrG XbaI R | CGGTCTAGACTGTCTGAGAGGAGGCACTGATGCG |
|  | thiAp SpeI F | CGCGACTAGTCGACCTGGCACCTACAGAAGAATC |
|  | thiAp SpeI R | CGCGACTAGTGTTGACTCAGTTCAATGGTTCGAC |
